# Technique of sentinel lymph node biopsy and lymphatic mapping during laparoscopic colon resection for cancer

**DOI:** 10.3332/ecancer.2008.60

**Published:** 2007-11-15

**Authors:** PP Bianchi, B Andreoni, M Rottoli, S Celotti, A Chiappa, M Montorsi

**Affiliations:** 1 Department of General and Laparoscopic Surgery, European Institute of Oncology IRCCS; 2 Department of General Surgery, Istituto Clinico Humanitas IRCCS, School of Medicine, University of Milano, 20141 Milano, Italy

## Abstract

**Background:**

The utility of lymph node mapping to improve staging in colon cancer is still under evaluation. Laparoscopic colectomy for colon cancer has been validated in multi-centric trials. This study assessed the feasibility and technical aspects of lymph node mapping in laparoscopic colectomy for colon cancer.

**Methods:**

A total of 42 patients with histologically proven colon cancer were studied from January 2006 to September 2007. Exclusion criteria were: advanced disease (clinical stage III), rectal cancer, previous colon resection and contraindication to laparoscopy. Lymph-nodal status was assessed preoperatively by computed tomography (CT) scan and intra-operatively with the aid of laparoscopic ultrasound. Before resection, 2–3 ml of Patent Blue V dye was injected sub-serosally around the tumour. Coloured lymph nodes were marked as sentinel (SN) with metal clips or suture and laparoscopic colectomy with lymphadenectomy completed as normal. In case of failure of the intra-operative procedure, an *ex vivo* SN biopsy was performed on the colectomy specimen after resection.

**Results:**

A total number of 904 lymph nodes were examined, with a median number of 22 lymph nodes harvested per patient. The SN detection rate was 100%, an *ex vivo* lymph node mapping was necessary in four patients. Eleven (26.2%) patients had lymph-nodal metastases and in five (45.5%) of these patients, SN was the only positive lymph node. There were two (18.2%) false-negative SN. In three cases (7.1%) with aberrant lymphatic drainage, lymphadenectomy was extended. The accuracy of SN mapping was 95.2% and negative predictive value was 93.9%.

**Conclusions:**

Laparoscopic lymphatic mapping and SN removal is feasible in laparoscopic colectomy for colon cancer. The *ex vivo* technique is useful as a salvage technique in case of failure of the intra-operative procedure. Prospective studies are justified to determine the real accuracy and false-negative rate of the technique.

## Context

The metastatic status of regional lymph nodes is a major prognostic factor in colon cancer and is particularly important for determining adjuvant therapy [1–4]. Five-year survival is 85–95% for stage I colon cancers, 60–80% for stage II, but 30–60% for stage III disease (characterized by nodal involvement) [5, 6]. Adjuvant chemotherapy confers a 33% survival benefit in patients with stage III disease [7, 8], but serves no purpose in patients without nodal involvement [9]. However, survival variability and high recurrence rates in stage II patients suggest that current lymph node staging procedures understage too many patients.

Key variables determining whether lymph node metastases are found (and what stage is assigned) are the number of nodes harvested [2, 3] and the method of node evaluation [4]. The probability of finding an involved node increases with number of nodes recovered, suggesting that all palpable nodes, even small ones, should be harvested [2]. Similarly, the conventional node examination method—single-level sectioning and haematoxylin and eosin (H&E) staining—may fail to detect tumour cells [4]. At the same time methods of lymph node ultrastaging, such as multi-level sectioning, immunohistochemistry and polymerase chain reaction, are costly and time-consuming particularly if applied to all nodes harvested. Sentinel lymph node (SN) mapping, developed to identify and examine the nodes at highest risk of metastatic involvement in patients with melanoma and breast cancer [10, 11], has been shown to improve staging in these diseases. The aims of SN mapping in colon cancer are to identify and examine the nodes with highest probability of metastatic involvement, and also to identify nodes not on conventional lymphatic tracts.

Laparoscopic surgery is a validated method for the treatment of colon cancer [12–14]; however, few publications have described SN mapping in laparoscopic colon resection [15–20]. The purpose of this paper is to describe technical aspects of SN mapping in laparoscopic colon surgery for colon cancer, and to assess its feasibility in an initial series of patients.

## Materials and Methods

### Patients

From January 2006 to September 2007 44 patients—26 men, 18 women, mean age 63 years (range 31–74) with clinical stages I and II colon cancer—were prospectively enrolled in the study. Exclusion criteria were metastatic disease, rectal cancer, advanced disease with invasion of adjacent structures, previous colonic resection and contraindications to laparoscopy. Five earlier cases, not included in the series, were used to gain experience with SN mapping. Exclusion criteria were metastatic disease, rectal cancer, advanced disease with invasion of adjacent structures, previous colonic resection and contraindications to laparoscopy.

Pre-operative work-up comprised abdominal and pelvic CT, chest x-ray and colonoscopy with biopsy. Lymph-nodal clinical stage was assessed preoperatively by CT scan and intra-operatively by a complete laparoscopic staging with laparoscopic ultrasound.

Lymph nodes detected by CT scan with a volume higher than 1.0 cm were considered positive. Patients with distant metastases, T4 disease or clinical-node-positive disease discovered during intra-operative staging were excluded (two patients of the present series). The lesion, identified during colonoscopy, was marked with china ink or metal clips for subsequent laparoscopic identification. The positions of any polyps removed during colonoscopy were also marked [21].

### Technique

All lymph node mapping procedures were performed by a single surgeon (PPB), experienced in SN detection. After pneumo-peritoneal insufflation to about 12 mmHg, complete laparoscopic disease staging was performed with the aid of laparoscopic ultrasonography [22]. After lesion location, insufflation pressure was reduced to 6 mmHg, the colonic segment containing the lesion was carefully manoeuvred to the abdominal wall with the aid of atraumatic graspers, and the site of percutaneous access was selected. Colon mobilization was not performed at this stage in order to maintain lymphatic tree integrity.

A 22-gauge spinal needle was inserted into the colon sub-serosally at an angle of about 45° to the tangential plane of the colon at the chosen injection site. Low insufflation pressure helped obtain the correct needle insertion angle. Care was taken to avoid inserting the needle too far (into the muscular layer). Patent Blue V dye (SALF, Bergamo, Italy) 2–3 ml was injected sub-serosally at 3–4 sites around the tumour. A large sponge was inserted into the abdominal cavity and positioned close to the needle, to mop up any dye leakage.

**Figure:**
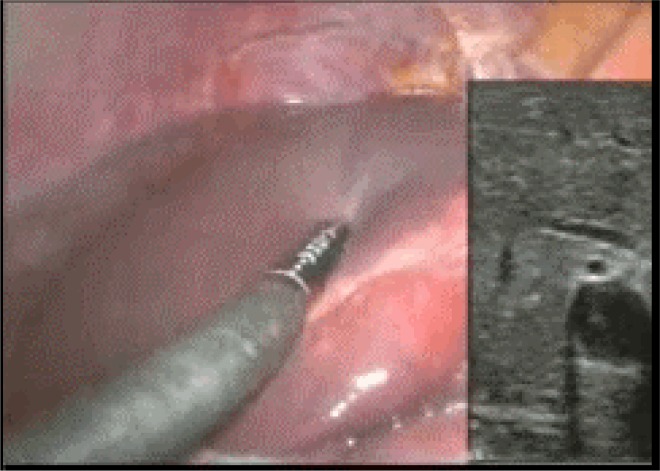
http://www.ecancermedicalscience.com/view-article.asp?doi=10.3332/ecancer.2008.60

Five to ten minutes were usually necessary to visualize the lymph ducts and the first node to receive dye. In obese patients, the mesenteric peritoneum was gently dissected to reveal the lymphatic path to a lymph node. The first lymph nodes to take up dye were considered SNs and marked with metal clips or a suture.

Care was taken to identify any coloured lymph nodes at unusual sites that would indicate the lymphadenectomy should be extended, as in a patient with a cancer of the right colon, where a blue lymph node was identified at the root of the transverse meso-colon.

Insufflation pressure was increased to 12 mmHg and the colectomy was completed with ligation of the mesenteric blood vessels at their origins and radical lymphadenectomy. In left colon resections, a medial-to-lateral technique was used, with complete splenic flexure mobilization and intra-corporeal transanal double-stapled anastomosis. For right colon resection, the anastomosis was performed extra-corporeally, via median periumbilical mini-laparotomy.

In four cases, *ex vivo* SN mapping was performed on the colectomy specimen immediately after resection, following the failure of intra-corporeal mapping. The procedure was similar to that used for intra-corporeal SN mapping. In all cases, marked SNs were removed from the resected specimen and sent to the pathologist separately.

## Results

In two of the 44 patients enrolled, SN mapping was not performed because the unexpected detection at the intra-operative staging of advanced disease. Thus SN mapping was successfully performed in 42 patients, 17 of whom had right colon and 25 had left colon lesions. The mean duration of the mapping procedure was 14 minutes (range 10–18).

Eight patients received pre-laparoscopic endoscopic polypectomy and the resections site(s) were marked with black ink. In seven patients, the lesions identified endoscopically were tagged with two metal clips, permitting the use of ultrasound for intra-operative tumour localization.

*Ex vivo* procedure was performed in four cases because of failure of intra-operative mapping. In all the cases, the cause of failure was due to technical difficulties, with spillage of the dye inside the lumen of the colon in one case and in another in the peritoneal cavity; in another patient, with a lesion located in the left colon, the left meso-colon and mesentery adhered together and it was not possible to complete the SN mapping; and in the last case, an obese patient with a body mass index of 49, with a small lesion of the right colon, the exact site of the lesion was not evident intra-operatively.

At least one SN was identified intra-corporeally in 38 patients. In the remaining cases, after the specimen had been removed, *ex vivo* mapping was performed successfully. A total of 904 lymph nodes were harvested with a median of 22 (range 8-–38) lymph nodes per patient. SNs identified were 93, an average of two (range 1–4) per patient. Eleven (26.2%) patients had positive lymph nodes and positive SNs were found in nine patients (21.4%). Thus, in two cases, the SNs were negative and non-SNs were positive, the false-negative rate was therefore 2/11 = 18.2% (Table 1). In five cases, the SN was the only positive node (possible upstage) and in 40 cases (95.2%) the SN correctly reflected nodal status. Isolated tumour cells were found in the SNs of two patients and cytokeratin immunohistochemistry was necessary to confirm metastasis.

Micro-metastasis was found in one SN. Thus three patients (9.6%) were truly upstaged by ultrastaging methods. The pathological staging of all patients is shown in Table 2.

In three (7.1%) patients, mapping revealed lymphatic drainage outside the planned margins of the resection. Two cases were right colon cancer, in one SN mapping revealed a blue node at the base of the transverse meso-colon, to the left of the middle colic artery, in the other, an SN was located near the small bowel wall at more than 10 cm from the ileocecal valve and in the third patient, with a left colon lesion, a blue node was revealed in the aortic bifurcation plane. In all these cases, the lymphadenectomy was extended to include the blue nodes. In all cases, SNs were negative at histopathologic examination, and disease stage did not change.

## Discussion

The main goal of SN mapping in colon cancer is to improve lymph-nodal staging to identify the number of patients classified as stage II, and varying from 20% to 30%, that will die within five years from tumour relapse or distant metastases and will thus benefit from adjuvant chemotherapy [4–6]. Furthermore, patients with advanced diseases will receive adjuvant chemotherapy anyway. Therefore the study of SNs should be directed only to these early stages.

Moreover an accurate selection of patients, excluding advanced diseases, would reduce the number of skip metastases and would increase the detection rate of SN mapping. In fact, in advanced diseases lymphatic flow may be altered by the closure of lymphatic ducts near the lesion. Therefore we decided to concentrate our SN study only on patients with a clinical stage I or II disease. These early stage patients are the class of patients that can benefit more from minimally invasive treatment, because the highest number of conversions from laparoscopy to laparotomy are in advanced cancer cases, with invasion of the adjacent structure and in the bulky diseases [23].

Few publications have described SN mapping in laparoscopic colon resection [15–20]. In all the studies, as in ours, lymphatic mapping was performed with blue dye injected sub-serosally, intra-operatively or sub-mucosally by intra-operative endoscopy. This is a quick (mean duration 14 minutes) and quite easy method. When technical difficulties are present and SN mapping fails, the *ex vivo* mapping is performed as a salvage technique. Some authors have performed only this *ex vivo* technique [24, 25], but in our opinion *in vivo* mapping has a relevant role. This is because it detects aberrant lymphatic drainage (7.1% in this series), avoids the interruption of the lymphatic pathway by any surgical manipulation, and because the direct visualization of lymphatic pathway, particularly in laparoscopic surgery, gives a clearer identification of the lymph nodes basin, helping the surgeon execute a radical lymphadenectomy.

Since 1999 numerous papers on the SN technique in colorectal cancer have been published, with a wide range of results in terms of accuracy and false negatives: False-negative rates in fact vary from 0% to 60% [26–36]. This astonishing range has several explanations. First the SN mapping technique has not been standardized; some authors inject blue dye sub-mucosally via an endoscopic approach; others prefer intra-operative sub-serosal injection; others again use radioactive tracer either injected sub-mucosally the day before surgery, or sub-serosally intra-operatively [37–39]. Use of fluorescein dye has been described with good results [40], and recently, an intra-operative near-infrared fluorescence imaging system has been reported that displays surgical anatomy and injected quantum dots by near-infrared fluorescence [41].

Saha ***et al*** [36] emphasized the importance of adequate training and use of a standardized technique to reduce technical problems, and this matches our own experience. We emphasize also that an adequate amount of dye, injected at 3–4 points around the tumour, is crucial for success. At the same time intra-luminal dye leakage must be avoided to prevent dye absorption (and subsequent diffusion) by areas distant from the primary. Viehl ***et al*** [42] also emphasized these points, noting that the injection of 1 ml of dye is insufficient to reliably identify SNs. We use 2–3 ml, and tend to use more in patients with a thick meso-colon.

Patient selection may influence the success rate of SN mapping and staging accuracy; large cancers and those with massive nodal involvement are likely to alter lymphatic flow by closing lymphatic ducts near the lesion and hence increase the false-negative rate. Furthermore, in advanced disease, SN study is less useful as patients will receive adjuvant chemotherapy anyway. In a recent multi-centric trial, a cut-off level of 22 patients was defined to increase detection rate from 76.4% to 91.0% [43].

A more intriguing problem is the prognostic significance of micro-metastases and isolated tumour cells (ITCs) in the SN. Some studies have found that patients with a clear SN have better survival than those with micro-metastases [44, 45]; other studies indicate that micro-metastases and ITCs have no prognostic significance [35, 46].

The prognostic implications of SN mapping for nodal staging and recurrence emerged clearly from the multi-centre SN mapping trial of Saha ***et al*** [36]. Among patients with a minimum of two years’ follow-up, they found a seven per cent recurrence rate in those undergoing SN mapping compared to a 25% recurrence rate in those receiving standard resection and nodal staging. Furthermore, among the 500 patients receiving SN mapping, metastases were detected in 50%, while among the 368 patients not mapped, nodal metastases were detected in only 35%. However, the Saha ***et al*** trial employed open surgery.

The experience of this study is that SN mapping is feasible, as it is a relatively brief procedure and identified at least one SN in all 42 cases. The achievement of this good result depended on the employment of standardized procedures learnt by experience, including reduction of intra-abdominal pressure during injection, injecting sub-serosally at an angle of about 45°, and mopping up any dye leakage with a large sponge. In our series, SN status reflected regional node status in 40 out of 42 cases giving an accuracy of 95.2%.

We harvested a median of 22 lymph nodes per patient; further indication that laparoscopic colectomy provides an oncologically adequate approach. In two patients (7.1%), the lymphadenectomy was extended to nodal stations not usually included in the resection, following identification of SNs outside the margins of the initially planned resection. Somewhat higher (29% and 28%) proportions of aberrant lymph drainage were reported by Tsioulias ***et al*** [16] and Bilchik and Trocha [18], while in both these studies less lymph nodes per patient were harvested.

The false-negative rate of 18.2% is the least satisfactory finding of our study; nevertheless, it is not of huge relevance, because these patients have been correctly staged, and have received adjuvant chemotherapy anyway.

Our overall experience in this study indicates that SN mapping in laparoscopic colectomy is feasible, with a high accuracy rate, and promising as a method of improving the nodal staging of stages I and II colon cancer. Larger prospective studies to determine the accuracy and false-negative rate of the technique as well as the prognostic role of micro-metastasis and ITC are justified.

## Figures and Tables

**Table 1:**
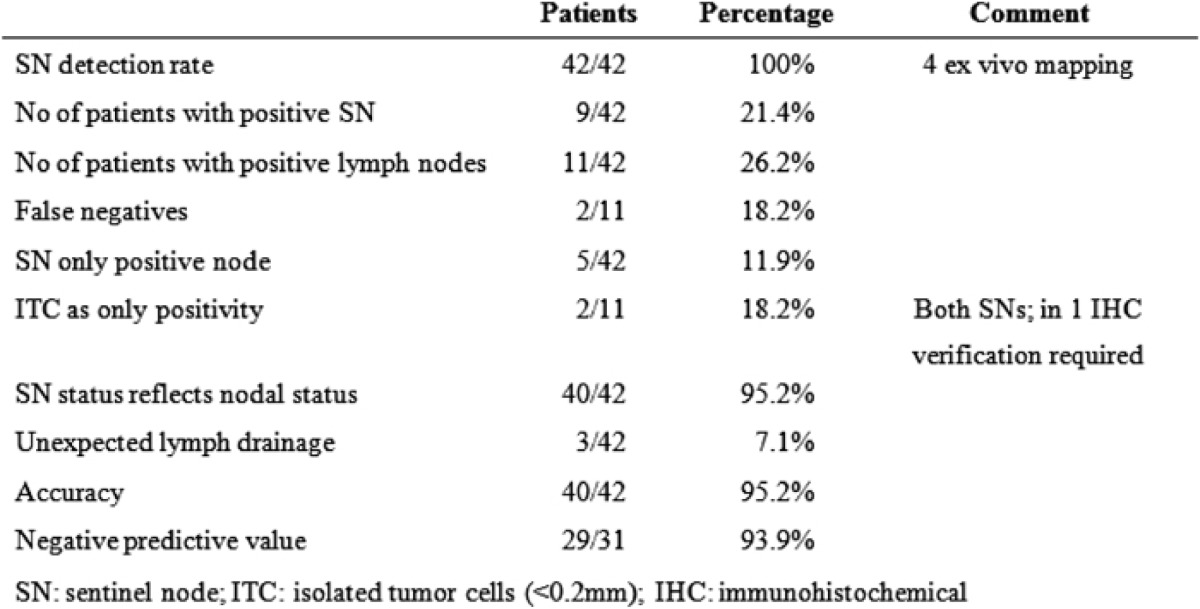
Results of sentinel node mapping in 42 patients undergoing laparoscopic colectomy for colon cancer

**Table 2:**
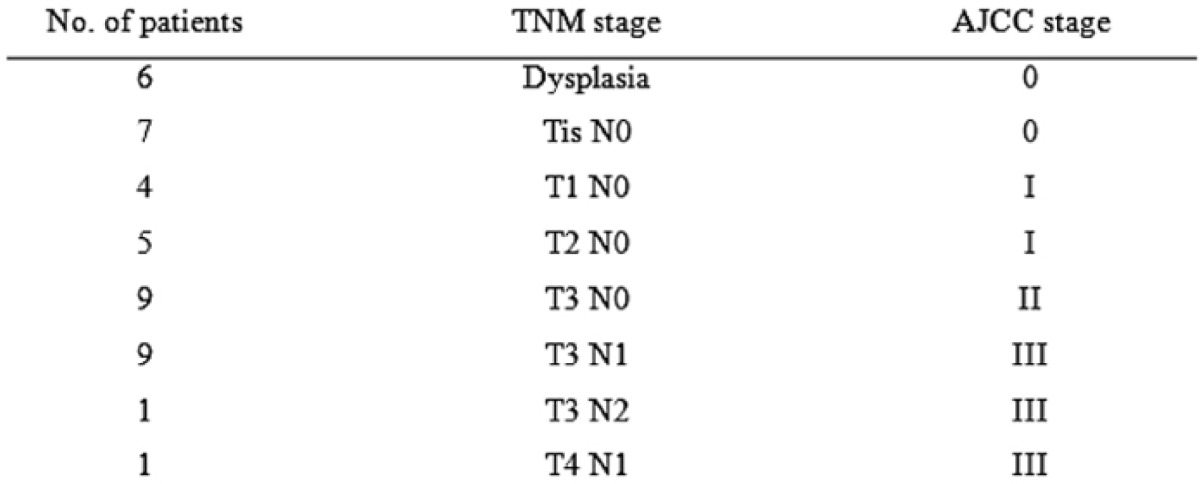
Pathological staging of 42 patients undergoing laparoscopic colectomy for colon cancer
